# There Is More Than One Kind of Extinction Learning

**DOI:** 10.3389/fnsys.2019.00016

**Published:** 2019-05-07

**Authors:** Jarid Goodman, Mark G. Packard

**Affiliations:** ^1^Department of Psychology, Delaware State University, Dover, DE, United States; ^2^Department of Psychological and Brain Sciences, Texas A&M University, College Station, TX, United States

**Keywords:** extinction, memory, learning, hippocampus, striatum, basal ganglia

## Abstract

The view that different kinds of memory are mediated by dissociable neural systems has received extensive experimental support. Dissociations between memory systems are usually observed during initial acquisition, consolidation, and retrieval of memory, however increasing evidence also indicates a role for multiple memory systems in extinction behavior. The present article reviews a recent series of maze learning experiments that provide evidence for a multiple memory systems approach to extinction learning and memory. Evidence is described indicating that: (1) the hippocampus and dorsolateral striatum (DLS) mediate different kinds of extinction learning; (2) the effectiveness of different extinction protocols depends on the kind of memory being extinguished; and (3) whether a neural system is involved in extinction is also determined by the extinction protocol and kind of memory undergoing extinction. Based on these findings, a novel hypothetical model regarding the role of multiple memory systems in extinction is presented. In addition, the relevance of this multiple memory systems approach to other learning paradigms involving extinction (i.e., extinction of conditioned fear) and for treating human psychopathologies characterized by maladaptive memories (e.g., drug addiction and relapse) is briefly considered.

## Introduction

“I wish to suggest that our familiar theoretical disputes about learning may perhaps (I emphasize ‘perhaps’) be resolved, if we can agree that there are really a number of different kinds of learning.”

*—Tolman (1949)*.

Extinction may be broadly defined as the learned suppression of a previously acquired memory (Bouton, 2004; Myers and Davis, 2007). When a subject is returned to a situation in which some memory had been acquired, but without the original reinforcer that had motivated initial acquisition of the memory, extinction learning typically follows. Extinction learning becomes evident when the behaviors that had manifested during initial acquisition of the memory begin to decline. For instance, a rat that had acquired a running approach response down a straight alley to retrieve food reward at the opposite end of the maze will demonstrate extinction learning when the food reward is later withdrawn, and extinction learning will be expressed behaviorally as a suppression of the original running approach response. Decrements of the original behavior constitute the most commonly cited outcome of extinction training and serve as the dominant measure of extinction learning and memory in most studies.

In the first half of the 20th century, extinction was interpreted in the context of learning theory, which at the time was dominated by two opposing viewpoints: the stimulus-response (S-R) view and the cognitive view. Clark L. Hull, who provided the most complete iteration of the S-R view at this time, suggested that initial acquisition of a memory may be likened to an acquired reflex, to the extent that stimuli (S) in the learning environment may gain the capacity to activate automatic behavioral responses (R; Hull, 1943). During extinction training, the opposite occurs and, instead of stimuli having an excitatory impact on the response, stimuli gain the capacity to activate a habit of not responding or a “no response.”

In opposition to the S-R view, the cognitive view championed by Tolman (1932) suggested that, during initial learning, animals acquire meaningful relationships between stimuli in the learning environment. These learned associations between stimuli culminate into a “sign-gestalt expectation” that guides behavior to the reinforcer (e.g., food reward). During extinction training, a change in expectation occurs in which the animal expects the *absence* of reinforcement. Therefore, to the extent that the expectation of reinforcement had guided the original behavior, expecting the *absence* of reinforcement during extinction training should result in a response decrement.

Although Tolman was a passionate advocate for the cognitive view of learning, he also offered the possibility that “there is more than one kind of learning” (Tolman, 1949). Tolman suggested that some of the debates between learning theorists could be resolved if we accepted that the distinct learning mechanisms being proposed by different groups are not mutually exclusive and that they instead co-exist and contribute uniquely to learning and memory function. Over the past few decades, this general contention has been extensively corroborated by neurobiological studies indicating that the acquisition and retrieval of different kinds of information are mediated by different parts of the brain (for review see Squire, 2004; White et al., 2013). That is, instead of a single mechanism guiding learning and memory, these processes may be achieved through multiple memory systems. However, dissociations between memory systems have been observed primarily during initial acquisition, consolidation, and retrieval of memory, whereas comparatively little attention has been devoted to a potential role for multiple memory systems in extinction learning.

**Figure 1:**
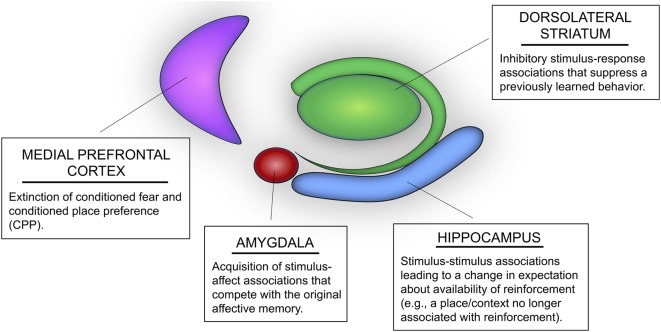
Potential roles of multiple memory systems in extinction learning and memory. The hippocampus mediates stimulus-stimulus associations that could lead to changes in expectation of reinforcement. For instance, extinction learning mediated by the hippocampus may include learning that a context or spatial location is associated with “no food” or “no shock.” The dorsolateral striatum (DLS) mediates inhibitory stimulus-response associations, whereby stimuli in the learning environment may be associated with inhibition of a previously acquired behavior (e.g., inhibition of a previously learned body-turn response or lever press). The basolateral amygdala (i.e., BLA) mediates new stimulus-affect associations that compete with previously acquired conditioned emotional memories, such as Pavlovian fear conditioning and conditioned place preference (CPP). Likewise, the medial prefrontal cortex (mPFC) has also been identified as an important region mediating extinction of conditioned fear and CPP.

The present article primarily reviews evidence from a recent series of maze learning experiments conducted in our laboratory and introduces a multiple memory systems approach to extinction (Figure 1). This approach suggests that there are different kinds of extinction learning that can contribute to the response decrement following extinction training. Moreover, different kinds of extinction learning may be achieved through distinct learning mechanisms mediated by different parts of the brain. The present article also considers: (1) how this multiple systems view can be applied to extinction in other learning paradigms, namely extinction of conditioned fear; and (2) how this approach might be relevant to suppression of maladaptive memory in neuropsychiatric disorders.

## Multiple Memory Systems: Hippocampus and Dorsal Striatum

The present review describes a multiple memory systems approach to extinction by providing evidence that the hippocampus and dorsolateral striatum (DLS) mediate different kinds of extinction learning. This idea is based in part on extensive evidence across several mammalian species indicating that these two structures are involved in the initial acquisition of different types of information (Packard et al., 1989; Packard and McGaugh, 1992, 1996; McDonald and White, 1993; Knowlton et al., 1996; Teng et al., 2000; Fernandez-Ruiz et al., 2001). The hippocampus selectively mediates stimulus-stimulus associations, which can be employed to build cognitive maps of the learning environment or “sign-gestalt expectations” about the learning situation (O’Keefe and Nadel, 1978). This information can be used to guide purposive behavior in the learning situation, such as directing maze running behavior toward a rewarded spatial location.

In contrast, the DLS mediates associations between stimuli and responses (for review see Packard and Knowlton, 2002), so that stimuli can automatically activate a behavioral response. Following acquisition, the learned behaviors inherent in an S-R memory mediated by the DLS are expressed without cognitive “expectation” of reinforcement, but rather are activated by specific stimuli. Notably, learning and memory functions of the DLS appear remarkably consistent with Hull’s S-R habit view of learning, whereas the mnemonic functions of the hippocampus resemble Tolman’s cognitive view of learning (Tolman, 1932; Hull, 1943).

Considering the extensive evidence that hippocampus and DLS are involved in distinct learning and memory processes guiding initial acquisition and retrieval, it is possible that these neural systems also subserve different kinds of extinction learning. Determining whether the DLS and hippocampus are involved in different kinds of extinction learning would require the use of separate extinction protocols that presumably engage distinct learning mechanisms.

## Two Protocols: Latent vs. Response Extinction

**Figure 2:**
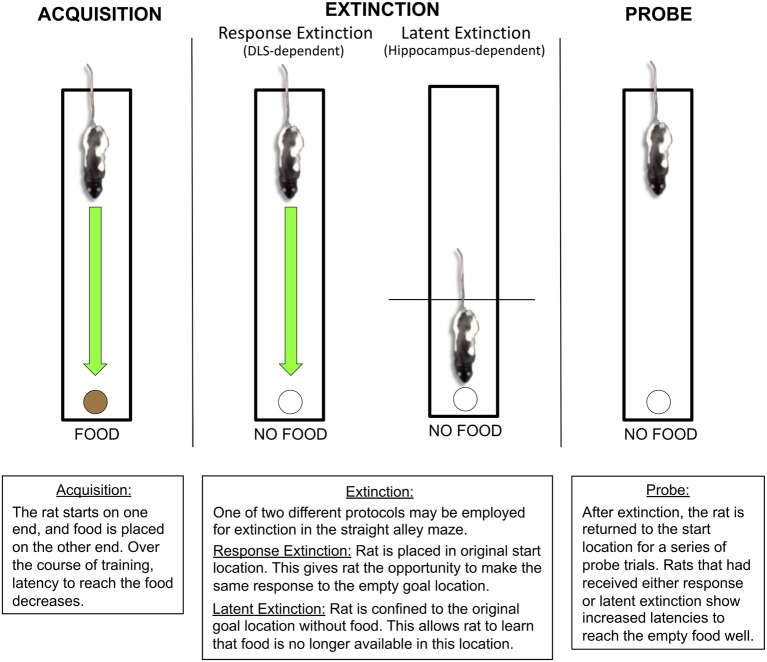
Latent and response extinction in the straight alley maze. During initial acquisition in the straight alley maze, the rat is consistently placed on one end of a straight runway, while food remains available at the other end in a recessed food well. Over the course of acquisition, the rat learns to make a running approach response to the food location. Following acquisition, behavior can be extinguished using one of two distinct protocols: response extinction or latent extinction. For response extinction, the rat is placed in the original starting location without food on the other end. This allows the animal to make the original running approach response to the empty food well. For latent extinction, the rat is confined to the goal location without food, which prevents the rat from making the original running approach response. The effectiveness of latent and response extinction is revealed through the use of subsequent probe trials, in which the animal is returned to the original starting position without food at the other end. During the probe trials, rats that had received either latent extinction or response extinction show an increase in latency to approach the food well, suggesting that both protocols lead to extinction. Research from our laboratory indicates that response and latent extinction are mediated by distinct memory systems (Gabriele and Packard, 2006; Goodman et al., 2016a; Goodman et al., 2017a). The DLS, but not the hippocampus, mediates response extinction. The hippocampus, but not the DLS, mediates latent extinction.

Early experimental psychologists demonstrated by training rats in a variety of maze tasks that extinction learning can be achieved using distinct protocols. In the straight alley maze (Figure 2), animals are initially trained to make a running approach response down a straight runway to retrieve food reward at the opposite end of the maze. Following initial acquisition of the straight alley maze, memory performance may be extinguished using two distinct protocols. In a typical “response extinction” protocol, a subject is given the opportunity to perform the original behavior, but without reinforcement. For example, response extinction in the straight alley maze involves releasing a rat from the original starting position, thus affording the animal the opportunity to execute the original running approach response toward the goal box at the opposite end of the maze, only now this goal box does not contain food.

On the other hand, a “latent extinction” protocol involves confining an animal to the previous goal location without reinforcement (Seward and Levy, 1949; Deese, 1951; Moltz, 1955; Denny and Ratner, 1959; Dyal, 1962; Clifford, 1964). Importantly, this extinction training protocol prevents the animal from having the opportunity to perform the originally acquired approach behavior. For example, latent extinction in the straight alley maze involves confining a rat to the goal box without food, thereby preventing the animal from performing the running approach response to the empty goal box. Even though the animal is not able to perform the originally reinforced running response, these goal box confinements remain effective at producing extinction by presumably “informing” the animal that the goal box no longer contains food (Seward and Levy, 1949). The effectiveness of latent extinction is revealed through subsequent probe trials, in which a rat is released from the original starting position, therefore having the opportunity to perform the running approach toward the unrewarded goal location. Animals previously given latent extinction demonstrate a greater suppression of the running approach response during these probe trials, relative to control animals that were confined to another, neutral box (Seward and Levy, 1949). These observations suggest that some extinction learning occurs during the unreinforced goal box confinements, i.e., without the animal having to perform the original running response.

Historically, latent extinction provided evidence against the S-R view, which hypothesized that the unreinforced response must be made in order for extinction to occur (Hull, 1943). Instead, latent extinction was viewed as providing evidence for the cognitive view, in that the response decrement may be attributed to a change in expectation about the goal location no longer containing reinforcement (Seward and Levy, 1949; but see also Moltz, 1955). Thus, latent extinction contributed in part to the downfall of the S-R view and the rise of the cognitive view of extinction (Hulse et al., 1975).

However, an alternative approach based on the multiple memory systems hypothesis is that latent and response extinction may tap into different kinds of learning mediated by distinct neural systems. Unreinforced confinements to the goal box during latent extinction training could allow animals to acquire a new association in which the originally rewarded place is associated with the absence of reinforcement. Indeed, latent extinction is most effective when conducted in the presence of extra-maze cues that are conducive to spatial memory processing (Seward and Levy, 1949; Bugelski et al., 1952; Scharlock, 1954; Denny and Ratner, 1959; Dyal, 1962). In addition, being confined to a neutral goal box in a different room or a distinct spatial location in the same room does not result in a response decrement commensurate with latent extinction (Iwahara et al., 1953; Clifford, 1964). In contrast to latent extinction, response extinction remains effective in the absence of allocentric spatial cues (e.g., Scharlock, 1954), suggesting that response extinction might depend on a distinct learning mechanism. One possibility is that animals given response extinction may acquire inhibitory S-R associations that suppress the original behavior, consistent with the Hullian S-R view.

It should be emphasized that whether response extinction specifically relies on Hullian S-R mechanisms and latent extinction relies on Tolmanian cognitive mechanisms has not been definitively demonstrated. However, these proposed mechanisms are partially supported and prove useful in generating hypotheses about what brain regions could be involved in latent and response extinction.

## Hippocampus and Dorsal Striatum Mediate Different Kinds of Extinction Learning

Considering that acquisition, consolidation, and retrieval of different kinds of memory have been associated with anatomically dissociable neural systems, different kinds of extinction learning could also be associated with distinct neurobiological substrates. Consistent with the potential role of inhibitory S-R mechanisms, response extinction could depend on the function of the DLS. In contrast, given the potential role of cognitive/spatial memory mechanisms, latent extinction could depend on the function of the hippocampus. Accordingly, the role of the DLS and hippocampus in response and latent extinction was examined in a series of experiments conducted in the straight alley maze (Figure 2). During initial acquisition of this task, animals were placed in a consistent starting position of a straight runway, and food reward was consistently located in a recessed food well at the opposite end of the runway. Over the course of initial acquisition, no drugs were administered, and mean latency to reach the food well decreased dramatically for all rats. For response extinction training, rats were placed in the original starting position and had the opportunity to make the original running approach response to the empty goal location. Animals receiving temporary DLS inactivation with bupivacaine prior to each response extinction session demonstrated lower latencies to reach the empty goal location, relative to animals given saline infusions (Goodman et al., 2017a). In contrast, hippocampal inactivation did not significantly influence running latencies during response extinction training (Gabriele and Packard, 2006). These findings suggest that the kind(s) of extinction learning invoked by the response extinction protocol depends on DLS activity, but not hippocampal activity.

In contrast, the hippocampus is required for latent extinction (Gabriele and Packard, 2006). For latent extinction training, animals were confined to the goal location without food. Immediately prior to each latent extinction session, animals received temporary dorsal hippocampal inactivation with bupivacaine or control injections of saline. Following latent extinction training, both groups received drug-free probe trials in which animals were returned to the original starting position, and latency to reach the empty goal location was recorded. Animals that previously received hippocampal inactivation during latent extinction training demonstrated lower extinction latencies than saline control animals during the probe trials, indicating an impairment in extinction learning (Gabriele and Packard, 2006). In contrast, animals having received DLS inactivation during latent extinction training displayed comparable extinction latencies to saline-treated control animals during the subsequent drug-free probe trials (Goodman et al., 2017a).

The findings from these experiments demonstrate a double dissociation regarding the role of multiple memory systems in extinction learning. The DLS, but not the hippocampus, is needed for response extinction, whereas the hippocampus, but not the DLS, is needed for latent extinction. One interpretation of these findings is that response and latent extinction protocols tap into different kinds of extinction learning, which are mediated by dissociable neural systems.

## Does the Memory System Used for Acquisition Matter?

One issue to consider with the straight alley maze task is that it remains unclear what kind of memory is initially acquired in the task, as several different types of learning could potentially contribute to acquisition. According to the Hullian S-R view, stimuli in the learning environment may have acquired the ability to activate the running approach response. On the other hand, according to Tolman’s cognitive view, animals may have acquired information concerning the spatial location of the food reward, and the running approach response was purposefully directed toward this location. Whether animals acquire an S-R memory, cognitive memory, or both in this task is difficult to determine, because acquisition of either type of memory would result in the same approach response behavior. Indeed, both the DLS and hippocampus have been implicated in initial acquisition in the straight alley maze (Dunnett and Iversen, 1981; Kirkby et al., 1981; Rawlins et al., 1985), suggesting that both S-R and cognitive mechanisms could be involved.

Because it remains unclear precisely what kind of memory was initially acquired in the straight alley maze, we also do not know what kind of memory was being extinguished. Consideration of the initially acquired memory leads to important empirical questions. For one, it is important to consider whether latent and response extinction protocols are effective at extinguishing all kinds of memory, or in contrast whether each of these protocols is only effective at suppressing a specific kind of memory. In addition, we should also consider whether the DLS and hippocampus might still be implicated in response and latent extinction when different kinds of memory are being extinguished.

## Effectiveness of Latent and Response Extinction Depends on the Kind of Memory Being Extinguished

Considering that latent and response extinction protocols may invoke different kinds of extinction learning, it remains possible that each of these protocols is only effective for certain kinds of memory. In an initial experiment (Goodman and Packard, 2015), rats were trained in a place learning version of the plus-maze task. Initial acquisition of place learning depends on spatial memory processing mediated by the hippocampus and not the DLS (Packard and McGaugh, 1996; Chang and Gold, 2003). Over the course of initial acquisition in this task, animals were released into a plus-maze from varying starting positions (N or S), and food reward was located in a consistent goal arm (E). Following initial acquisition of the place learning task, separate groups of animals were given response extinction, latent extinction, or no extinction, and all groups were subsequently given probe trials to determine the effectiveness of these protocols. The number of perseverative trials (i.e., trials in which rats went directly to the previously rewarded location) and the latency to reach the previously reinforced spatial location were recorded. During the probe trials, animals previously given latent or response extinction displayed less perseveration and higher extinction latencies, relative to “no extinction” control animals, suggesting both protocols were effective at extinguishing the hippocampal memory.

In a second experiment (Goodman and Packard, 2015), separate groups of rats were trained in a response learning version of the plus-maze that depends on the function of the DLS and not the hippocampus (Packard and McGaugh, 1996; Chang and Gold, 2003, 2004). In this task, animals were again released from varying starting positions (N or S), however, acquisition of a consistent body-turn response (e.g., always turn left) was required in order to obtain the food reward. Following initial acquisition, animals were given response extinction, latent extinction, or no extinction. During subsequent probe trials, the response extinction group demonstrated less perseveration and higher latencies, relative to rats given no extinction (Goodman and Packard, 2015). In contrast, latent extinction did not significantly influence perseveration or running latencies, relative to the controls. These results suggest that response extinction, but not latent extinction, was effective at producing extinction of DLS-dependent response learning. The relative effectiveness of latent and response extinction training has also been demonstrated in water maze versions of the place and response learning tasks (Goodman et al., 2016a).

These findings provide evidence for a dissociation regarding the effectiveness of extinction protocols at targeting different kinds of memory. As latent extinction may involve an association between the original goal location and absence of reinforcement, this latent extinction memory could effectively interfere with the original memory acquired in the place learning task (i.e., memory that a spatial location contains reinforcement). However, following acquisition in the response learning task, learning that a spatial location does not contain reinforcement should be irrelevant to the original response learning memory. This is because memory performance in the response learning task is not guided by the spatial location of the reinforcement, but rather the execution of an egocentric turning response (Packard, 2009). On the other hand, the view that response extinction results in the formation of an inhibitory S-R association could explain why this extinction protocol was effective across both place and response learning tasks.

The above experiments suggest that within the context of the multiple memory systems view of extinction, the kind of memory being extinguished is an important factor to consider. However, these findings do not address the relative involvement of the hippocampus and DLS in extinction of different kinds of memory. In the following section, experiments are described suggesting that the involvement of a neural system in extinction learning might not only depend on the extinction protocol, but also the kind of memory being extinguished.

## Involvement of Neural Systems in Extinction Depends on Extinction Protocol and Kind of Memory Being Extinguished

An experiment was conducted to determine whether the hippocampus is still implicated in latent extinction when a hippocampus-dependent spatial memory is being selectively targeted (Goodman et al., 2016a). Animals were trained in a water-maze version of the place learning task described above (Schroeder et al., 2002). Following initial acquisition, rats were given latent or response extinction training. Intra-hippocampal injections of the NMDA receptor antagonist AP5 before latent extinction training were associated with lower extinction latencies during the subsequent drug-free probe trials, relative to saline-treated controls (Goodman et al., 2016a). However, animals previously given intra-hippocampal AP5 before response extinction training demonstrated comparable latencies to the saline-treated controls. These findings indicate that NMDA receptor activity in the hippocampus may be required for latent extinction of place learning.

In a separate series of experiments (Goodman et al., 2016b, 2017c), the role of the DLS in response extinction of different kinds of memory was examined. Rats were initially trained in appetitive versions of the DLS-dependent response learning task or the hippocampus-dependent place learning task, and subsequently received response extinction training. Immediately following the first day of extinction training, animals received post-training DLS inactivation with bupivacaine. Post-training drug infusions target initial consolidation of the extinction memory. Thus, whether post-training DLS inactivation disrupted consolidation of the extinction memory should become evident on the following day of extinction training. Results indicated that post-training DLS inactivation impaired memory consolidation of extinction in the response learning task, but not in the place learning task (Goodman et al., 2016b).

Interestingly, in the place learning task, DLS inactivation was associated with a significantly lower number of perseverative trials, relative to controls, suggesting an enhancement in extinction of place learning. Thus, the DLS may interfere with extinction in the place learning task, and thus removal of this interference leads to enhanced extinction. This interpretation invokes the idea that in some learning situations memory systems may compete with each other, in that disrupting the function of one memory system may lead to enhanced function of the other (for reviews, see Poldrack and Packard, 2003; Packard and Goodman, 2013).

To further examine the mechanism through which the DLS mediates extinction of response learning, animals were trained in the appetitive response learning task, and the potential role of DLS NMDA receptors was examined (Goodman et al., 2017c). Intra-DLS administration of the NMDA receptor antagonist AP5 impaired extinction memory, whereas intra-DLS administration of the NMDA receptor agonist D-cycloserine *enhanced* extinction memory in the response learning task.

In sum, the kind of memory being extinguished and the protocol used for extinction can determine what neural system will be needed for successful extinction learning. Hippocampus NMDA receptors mediate latent extinction, but not response extinction, of place learning. On the other hand, the DLS has been critically implicated in response extinction of a DLS-dependent response learning task, but not response extinction of a hippocampus-dependent place learning task. The role of the DLS in extinction of response learning may be partially attributed to activation of DLS NMDA receptors. Thus, NMDA receptor-dependent forms of synaptic plasticity in the hippocampus and DLS may be critical neural mechanisms supporting extinction of place learning and response learning, respectively. This mirrors the critical role of NMDA receptors in initial acquisition of hippocampal and DLS memories (Packard and Teather, 1997; Palencia and Ragozzino, 2005).

## The Multiple Memory Systems Approach to Extinction: A Hypothetical Model

The experiments described above provide evidence for a multiple memory systems approach to extinction. According to this approach, each extinction protocol engages a unique pattern of neural activity. Some extinction protocols might engage multiple neural systems equally, whereas other protocols might engage one neural system more than another. Latent extinction engages the hippocampus over the DLS, whereas response extinction engages the DLS over the hippocampus. However, it could also be argued that response extinction protocols, especially in spatial memory tasks, might also summon function of the hippocampus, albeit to a lesser degree. Consistent with this hypothesis, measures of hippocampal activity correlate with response extinction in some spatial memory tasks (Toumane et al., 1987, 1988; Topic et al., 2008; Porte et al., 2011). The observation that hippocampal inactivation does not influence the effectiveness of response extinction training might be attributed to another neural system (e.g., the DLS) being sufficient to produce the response decrement.

During an extinction protocol, the engaged neural system or systems mediate a unique kind of extinction learning. Whether that extinction learning is effective depends on the kind of memory being extinguished. A kind of extinction learning may be effective when it produces an extinction memory that competes with the original memory that guided behavior, whereas a kind of extinction learning may fail to be effective when the extinction memory is irrelevant to the originally acquired memory. Latent extinction presumably engages the hippocampus, which promotes a kind of extinction learning in which the original goal location is associated with absence of reinforcement. This new extinction memory may effectively compete with an original memory in which the spatial location was originally associated with presence of reinforcement (i.e., place learning), but would be ineffective at extinguishing memories that do not involve the spatial location of reinforcement (i.e., response learning).

The type of memory being extinguished might also determine whether a particular neural system is required for extinction. As mentioned above, the kind of memory acquired during initial acquisition of a task may only be extinguished by specific kinds of extinction learning. Thus, we should expect that inactivating the neural system that mediates the kind of extinction learning required would prevent extinction of that particular memory. For instance, evidence suggests that the kind of extinction learning invoked by a response extinction protocol (presumably an inhibitory S-R memory) may be needed for extinction of response learning, and that this kind of extinction learning depends on DLS activity (Goodman et al., 2017c). Thus, inactivation of the DLS blocks extinction of response learning (Goodman et al., 2016b).

However, the DLS is not required when using the response extinction protocol to extinguish a place learning memory. It is possible that a second kind of extinction learning might be invoked by the response extinction protocol. This other kind of extinction learning does not depend on DLS function and is sufficient to produce extinction of place learning. One possibility is that this second kind of extinction learning might involve a learned association between the original spatial location and the absence of the reinforcer (like latent extinction), and could be dependent on hippocampal function. Examination of this hypothesis would require the use of hippocampal inactivation during response extinction of place learning.

In sum, the multiple memory systems approach to extinction proposed here suggests that each extinction protocol engages a unique pattern of neural activity, sometimes engaging multiple neural systems equally and at other times engaging one neural system more than another. Each neural system mediates a unique kind of extinction learning involving distinct learning mechanisms. The effectiveness of a particular kind of extinction learning depends on the kind of memory being extinguished. Whether a neural system is required for extinction of a particular kind of memory depends on whether that neural system is mediating the kind of extinction learning responsible for suppression of the memory. Although this multiple memory systems approach to extinction has been discussed exclusively within the context of maze learning experiments, it might also be useful in understanding extinction in other learning situations.

## Multiple Memory Systems in Extinction of Conditioned Fear

In recent years, extinction has been extensively studied using fear conditioning paradigms (Milad and Quirk, 2012; Dunsmoor et al., 2015; Maren and Holmes, 2016). During initial acquisition of conditioned fear, a discrete stimulus (e.g., a tone) or context is repeatedly paired with an aversive foot-shock. When the tone or context is subsequently presented without foot-shock, the animals show a conditioned freezing response (CR), indicating that the animals had acquired a Pavlovian association between the tone (the conditioned stimulus; CS) or context and the foot-shock (the unconditioned stimulus; US). Extinction of conditioned fear may occur over time when the CS is repeatedly presented without the US, and is evidenced by a decrement in the CR. Fear extinction also involves the function of multiple memory systems. The primary brain regions investigated within the context of fear extinction include the amygdala, hippocampus, and medial prefrontal cortex (mPFC; Maren et al., 2013).

The amygdala has been classically considered as an important brain region involved in emotional behavior (Klüver and Bucy, 1939), and extensive research conducted over the past several decades has demonstrated that the amygdala also has a prominent role in emotional learning and memory processes. The basolateral complex of the amygdala (BLA) is principally implicated in the emotional modulation of long-term memories mediated by other brain regions (e.g., the hippocampus and DLS; McGaugh, 2004; Packard and Goodman, 2012; Goodman et al., 2017b). In addition, the amygdala has been regarded as the chief neural structure of an emotional memory system that mediates stimulus-affect associations (White and McDonald, 2002). In particular, the BLA mediates acquisition and expression of emotional memories that underlie Pavlovian fear conditioning (Phillips and LeDoux, 1992; Kim and Davis, 1993; Gale et al., 2004). Moreover, the BLA is also critically implicated in fear *extinction*. Blocking activation of NMDA receptors, metabotropic glutamate receptors, or the MAPK/ERK pathway in the BLA disrupts extinction of conditioned fear (Herry et al., 2006; Kim et al., 2007; Sotres-Bayon et al., 2007). Similarly, immediate early gene expression and protein synthesis in the BLA have also been associated with fear extinction (Lin et al., 2003; Herry and Mons, 2004). It should be noted that the BLA similarly mediates extinction of conditioned place preference (CPP) for drug rewards (e.g., Schroeder and Packard, 2003; Schroeder et al., 2002; Botreau et al., 2006; Sun and Laviolette, 2012). Thus, the amygdala may have a general role in extinction of stimulus-affect associations across multiple learning situations.

Prior research has also indicated an important role for the mPFC in fear conditioning. Specifically, the prelimbic cortex (PL) and infralimbic cortex (IL) of the mPFC are believed to play opposing roles in fear learning, in which the PL *activates* and the IL *suppresses* fear (for review see Giustino and Maren, 2015). Consistent with this model, there is evidence that the IL supports fear extinction (Quirk et al., 2006; Quirk and Mueller, 2008; Milad and Quirk, 2012). Inhibiting protein synthesis or NMDA receptor activation in the IL impairs the consolidation of fear extinction (Santini et al., 2004; Burgos-Robles et al., 2007). In addition, IL neurons show potentiated activity during extinction retrieval (Milad and Quirk, 2002). Evidence suggests that the IL promotes fear extinction *via* its projections to the amygdala. Specifically, the IL activates amygdala intercalated neurons, which in turn inhibit activity of the central amygdala, and this results in a suppression of the conditioned freezing response (Royer and Paré, 2002; Quirk et al., 2003; Berretta et al., 2005). Importantly, the mPFC also plays a role in extinction of CPP (Hsu and Packard, 2008), suggesting that the mPFC, like the amygdala, may have a general role in extinction of stimulus-affect memory.

The multiple memory systems approach to extinction described in this article may be helpful for understanding certain features of fear extinction. Investigators have advanced opposing views regarding what precise learning mechanisms underlie extinction of conditioned fear (for review see Dunsmoor et al., 2015). However, similar to what has been proposed for extinction of maze learning, it is possible that these proposed mechanisms are not mutually exclusive, and that different kinds of learning may potentially contribute to fear extinction. These kinds of extinction learning could be mediated by distinct brain regions and may be selectively engaged *via* different extinction protocols.

For instance, extensive evidence indicates that fear extinction is often associated with the context in which extinction training took place (Bouton, 2004). That is, when the animal successfully undergoes fear extinction in one context and is then moved to a different context, conditioned freezing reappears. This contextual renewal may be the result of one kind of extinction learning, i.e., where the context plays a major role in the suppression of freezing. Evidence indicates that this kind of extinction learning may be prevented by changing the parameters of the extinction protocol (e.g., Denniston et al., 2003; Monfils et al., 2009). In addition, the context dependency of fear extinction may be eliminated following hippocampal lesions (Wilson et al., 1995; Hobin et al., 2006), allowing animals to express fear extinction in multiple contexts. The fact that fear extinction continues to be expressed following hippocampal lesions, albeit with greater generalization, suggests that other brain regions (e.g., the BLA and IL) may contribute to fear extinction *via* different learning mechanisms, consistent with a multiple memory systems view of extinction.

## Multiple Memory Systems Approach to Suppression of Maladaptive Memory

An important therapeutic goal of animal models of extinction is to potentially adapt them to suppress maladaptive memories in human neuropsychiatric disorders (Powers et al., 2010; Milad and Quirk, 2012; Vervliet et al., 2013). In this regard, a multiple memory systems approach to extinction may be useful in several ways. The studies reviewed above indicate that the effectiveness of an extinction protocol depends on the kind of memory being extinguished. Specifically, an extinction protocol may only be effective when the novel memory acquired during extinction training competes with the original to-be-extinguished memory. Thus, it may be inferred that a reasonable strategy for treatment of human psychopathology is to select an appropriate behavioral protocol that targets the maladaptive memory.

As indicated above, response extinction, but not cognitive-based latent extinction, is effective at extinguishing DLS-dependent habit memory (Goodman and Packard, 2015; Goodman et al., 2016a). Thus, if a maladaptive behavior is governed by habit memory processes, cognitive/declarative information about the dangers or irrationalness of the behavior may do little in the way of extinction. Cigarette smokers are generally aware of the dangers of smoking, yet struggle to quit. People with OCD continue to perform ritualistic compulsions despite their understanding that the behavior is based on irrational obsessions (e.g., Rapoport, 1988). Habit-like symptoms in drug addiction, OCD, and other disorders have been associated with maladaptive dorsal striatum-dependent memory processes (Graybiel and Rauch, 2000; Everitt and Robbins, 2005; Marsh et al., 2005; Schwabe et al., 2011; Goh and Peterson, 2012; Goodman et al., 2012, 2014; Gillan and Robbins, 2014; Goodman and Packard, 2016), which could explain why these symptoms persist in the face of declarative information that the behavior is dysfunctional or potentially dangerous. It is possible that, like habit memory in rodents, habitual behavior in human psychopathology may be relatively resistant to cognitive forms of extinction, whereas behavioral treatments modeled after response extinction may be more effective. In rodents, cognitive latent extinction is less effective when the original behavior is reinforced with cocaine, relative to sucrose, whereas response extinction remains equally effective for cocaine or sucrose (Gabriele et al., 2009). Whether treatment of habitual behaviors in psychopathology may also predominantly benefit from response extinction procedures is an important area for future study.

Although investigations into the neurobiology of human psychopathologies often focus on a single neural locus or circuit underlying the pathology, it is more likely that these disorders involve multiple neural systems. PTSD, for instance, involves a neural circuit commonly implicated in fear learning (for review see Garfinkel and Liberzon, 2009). However, there is accumulating evidence that PTSD may also be associated with the function of the DLS habit memory system (Goodman and McIntyre, 2017; Rangaprakash et al., 2017), which could in part explain high rates of comorbid drug use and alcoholism in PTSD (for review see Goodman et al., 2012). The diversity of symptoms observed in PTSD, as well as those of other psychopathologies, may arise from multiple neural systems that contribute to the pathology in unique ways. From the point of view that a single extinction procedure may be limited in its ability to suppress different types of memory, there could be a therapeutic benefit to treating psychopathology with multiple extinction protocols, each designed to combat distinct symptoms of the disorder.

## Conclusion

Extinction learning can be considered within the context of a multiple memory systems framework. This approach suggests that: (1) different extinction protocols engage a distinct neural substrate (e.g., latent extinction engages hippocampal activity, whereas response extinction engages DLS activity); (2) the distinct pattern of neural activity associated with an extinction protocol produces a unique kind of extinction learning, characterized by distinct learning mechanisms (e.g., changes in expectation, inhibitory S-R associations, etc.); (3) whether a kind of extinction learning is effective depends on the kind of memory being extinguished; and (4) whether a neural system is implicated in extinction depends on not only the extinction protocol, but also the kind of memory undergoing extinction. The multiple memory systems approach may be useful for gaining a comprehensive understanding of extinction learning across multiple learning situations and also for tailoring behavioral and pharmacological treatments to alleviate specific kinds of maladaptive memory.

## Author Contributions

JG and MP contributed to the manuscript.

## Conflict of Interest Statement

The authors declare that the research was conducted in the absence of any commercial or financial relationships that could be construed as a potential conflict of interest.
